# Higher electronic health record burden among women physicians in academic ambulatory medicine

**DOI:** 10.1093/jamiaopen/ooaf164

**Published:** 2025-12-17

**Authors:** Sarah Y Bessen, Sean Tackett, Kimberly S Peairs, Lisa Christopher-Stine, Charles M Stewart, Lee D Biddison, Maria Oliva-Hemker, Jennifer K Lee

**Affiliations:** Department of Otolaryngology, Head & Neck Surgery, Johns Hopkins University, Baltimore, MD 21287, United States; Department of Medicine, Johns Hopkins University, Baltimore, MD 21287, United States; Biostatistics, Epidemiology, and Data Management Core, Johns Hopkins University, Baltimore, MD 21287, United States; Department of Medicine, Johns Hopkins University, Baltimore, MD 21287, United States; Johns Hopkins Sidney Kimmel Comprehensive Cancer Center, Baltimore, MD 21287, United States; Department of Medicine, Johns Hopkins University, Baltimore, MD 21287, United States; Department of Otolaryngology, Head & Neck Surgery, Johns Hopkins University, Baltimore, MD 21287, United States; Department of Medicine, Johns Hopkins University, Baltimore, MD 21287, United States; Office of Well-Being, Johns Hopkins University, Baltimore, MD 21287, United States; Department of Pediatrics, Johns Hopkins University, Baltimore, MD 21287, United States; Office of Faculty, Johns Hopkins University, Baltimore, MD 21287, United States; Office of Faculty, Johns Hopkins University, Baltimore, MD 21287, United States; Department of Anesthesiology and Critical Care Medicine, Johns Hopkins University, Baltimore, MD 21287, United States

**Keywords:** electronic health record, gender, burnout

## Abstract

**Objectives:**

Electronic health record (EHR) work may differently affect women and men physicians. Identifying gender discrepancies in EHR work across different specialties may inform strategies to reduce EHR burdens.

**Materials and Methods:**

We retrospectively evaluated EHR use by ambulatory physicians in 4 specialties (2 procedural [cardiology and gastroenterology] and 2 nonprocedural [internal medicine and rheumatology]) during 1 year at a large academic medical institution. Gender differences in EHR and clinical workload across specialties were evaluated by analysis of variance. Mixed-effects linear regression models analyzed gender differences in EHR workload controlling for specialty. Significant differences were additionally examined by stratifying procedural and nonprocedural specialties.

**Results:**

Clinical and EHR workload varied across specialties (*P* <.05), though scheduled clinical workload did not differ by gender. Controlling for specialty, women physicians spent more time per appointment on In Basket messages (*P* =.001), sent more Secure Chat messages per appointment (*P* =.003), and spent more time in the EHR outside 7:00 AM-7:00 PM (*P* <.001) than men. Gender differences in messaging were concentrated among the procedural physicians. Women procedural physicians spent more time on In Basket messages (*P* <.001) and sent more Secure Chat messages (*P* =.007) than men, whereas these differences did not occur among nonprocedural physicians.

**Discussion:**

Women physicians had greater EHR burdens despite similar scheduled clinical workloads as men. The greater messaging workload predominantly affected women procedural physicians.

**Conclusion:**

Gender disparities in EHR burden in ambulatory specialties vary between procedural and nonprocedural fields. Future research is needed to mitigate gender inequity in EHR workloads.

## Introduction

Retaining highly qualified physicians is one of the highest priorities in medicine due to physician shortages.^1^^,^^2^ The widespread adoption of the electronic health record (EHR) has drastically changed health-care delivery. While EHRs streamline clinical workflow and improve patient safety,^3^ they can also worsen work–life balance and burnout.^4–6^

Women comprise nearly half of clinical faculty in the United States,^7^ and gender influences whether physicians stay within or leave medicine as a profession.^8^ Although burnout affects all genders, women physicians report greater burnout than men.^4^^,^^5^ Physician burnout is associated with poorer quality of life,^9–11^ higher health system costs,^12^^,^^13^ greater risks of compromising patient care and safety,^14–18^ and higher physician turnover.^5^

Gender differences in communication may contribute to different EHR workloads. For example, compared to men, women pediatricians^19^ and gynecologists^20^ spend more time using a patient-centered communication style during clinical appointments. Different communication styles could influence a patient’s willingness to provide information or ask follow-up questions, thereby prompting more electronic correspondence, orders, and prescriptions.

The EHR burden may also vary between procedural and nonprocedural ambulatory specialties. Clarifying the types of EHR work with the greatest gender discrepancies in different specialties would inform strategies to optimize resource allocation and reduce excessive EHR work. Studies^21–25^ show higher EHR workload for women physicians but with limited information about which specialties have the largest gender inequities and thus are in greatest need of revised workflow. Moreover, because EHR use differs among institutions, gender discrepancies might be limited to specific locations or the inequity could be widespread.

As such, we investigated the associations between physician gender, EHR workload and patterns of use. We tested the null hypothesis that women and men physicians spend similar amounts of time electronically communicating with patients, other clinicians, and staff; receive and send a similar number of messages through the EHR; work in the EHR at similar times of the day and evening; and spend a similar amount of time writing orders and prescriptions. We secondarily compared gender differences in EHR burden in data stratified by procedural and nonprocedural specialties.

## Methods

### Study design, participants, and setting

We conducted a retrospective cross-sectional study on EHR use (Epic; Epic Systems, Verona, WI, United States) by faculty physicians in 4 specialties in ambulatory settings (2 procedural and 2 nonprocedural) at Johns Hopkins Medicine (Baltimore, MD). The procedural specialties were cardiology and gastroenterology, and the nonprocedural specialties were internal medicine and rheumatology. We chose these specialties a priori because they have a relatively balanced proportion of women and men physicians at our institution. The study was deemed exempt by the Johns Hopkins Institutional Review Board, and the requirement for informed consent was waived. We obtained data about EHR use from Signal, a provider efficiency tracking tool by Epic Systems, for all full-time faculty physicians who billed for ambulatory clinic services in one of the specialties between June 1, 2022, and July 31, 2023 (after clinical operations returned to baseline after the COVID-19 pandemic) and according to the Epic Signal inclusion criteria.^26^

We obtained the physician’s self-identified gender and verification of their full-time faculty status from the Johns Hopkins School of Medicine’s Office of Faculty Information. The study excluded physicians who were not faculty (such as clinical associates) or who were not employed full time (≥37.5 h/week) at Johns Hopkins Medicine.

### Variables

We extracted metrics of EHR use from Signal^27^ that are similar to those published by Sinsky et al.^28^ We measured overall workload by (1) the number of clinical appointments, (2) scheduled clinical hours per day, (3) the number of scheduled clinical days, (4) the number of days the physician logged into the EHR, and (5) the minutes spent conducting clinical review per appointment.

In Basket is Epic Systems’ communication hub for clinicians to receive and respond to messages from patients, notifications, results, and other types of communication. Physician electronic correspondence with patients, other clinicians, and staff members was assessed by (1) the number of In Basket messages received each day the physician was logged into the EHR, (2) minutes spent working in the In Basket per clinical appointment, (3) seconds to complete an In Basket message, and (4) turnaround time (days) to respond to an In Basket message. The amount of time the physician spends working on their In Basket reflects the total work involved in reading and responding to electronic communications. The number of seconds to complete an In Basket message indicates how quickly the physician answers a message once they have opened it. The turnaround time indicates how long it took for the physician to answer a message after it was sent by a patient, clinician, or staff member.

We measured additional electronic communications with other clinicians and staff by the number of messages sent through Secure Chat per appointment. Secure Chat is Epic Systems’ instant messaging system. We also measured the minutes a physician spent working on orders and prescriptions in the EHR per appointment.

Two variables measured how often physicians worked in the EHR outside typical working hours. First, Signal exported the minutes the physician spent working in the EHR outside of 7:00AM-7:00 PM per scheduled clinical day. Second, the “time outside of scheduled hours per scheduled day” quantified how long the physician worked in the EHR outside of their scheduled clinic hours, excluding the 30 min before their first and after their last appointments.

### Data analysis

We analyzed data normalized to clinical appointment or day and performed descriptive statistics for all variables. Differences in workload across the 4 specialties were evaluated by analysis of variance (ANOVA). Since the analogous nonparametric Kruskal–Wallis test did not show meaningful differences from the ANOVA, we report the ANOVA’s results. Because specialties have different patient populations and practice environments, to account for clustering of variance within specialties, we used separate mixed-effects linear regression models using each workload variable as the dependent variable, gender as a fixed effect, and specialty as a random. A Bonferroni correction was applied to adjust for multiple statistical comparisons. Thus, we considered an adjusted significance threshold of *P* < .004 (*α* = 0.05/14 comparisons) statistically significant.

For EHR metrics that reached statistical significance in the mixed-effects linear regression models, we stratified data by procedural and nonprocedural specialties to assess for gender differences using *t*-tests. Since the analogous nonparametric Wilcoxon rank-sum test did not show meaningful differences, we report the *t*-test results. Because the number of comparisons was small for this part of the analysis (3 comparisons for the procedural and 3 comparisons for the nonprocedural specialties), the significance threshold was not adjusted. The effect size, the standardized difference between means, was estimated using Cohen’s *d*. Effect sizes were considered small (*d* < 0.2), medium (*d* = 0.5), or large (*d* > 0.8).^29^ Analyses were performed using Stata version 13.0 (StataCorp, College Station, TX, United States) and graphs were generated using GraphPad Prism 9.4.1 (Boston, MA, United States).

## Results

We included a total of 187 faculty physicians (87 [47%] women) in the analysis. All physicians self-identified as either a woman or a man. One-hundred and eleven physicians were from procedural specialties (44 women [40%]). Seventy-six physicians were from nonprocedural specialties (43 women [57%]).

The 4 specialties had 10 differences from 14 metrics of EHR and clinical workload (Table 1). These included differences in the number of clinical appointments per day, the numbers of In Basket messages received and Secure Chat messages sent, and the amount of time spent on each In Basket message. The amount of time physicians worked in the EHR outside the 7:00 AM-7:00 PM period and the number of clinical appointments during the study period did not differ among the specialties.

**Table 1.** Physician EHR^a^ and clinical workload in 4 medical specialties in ambulatory settings.

	Procedural specialties		Nonprocedural specialties		
	1	2	3	4	*P*-value^b^
	Mean (SD)	Mean (SD)	Mean (SD)	Mean (SD)	
Scheduled workload					
Appointments during study period	643 (624)	683 (605)	672 (451)	681 (488)	.98
Clinic days	81 (61)	76 (45)	111 (54)	102 (40)	.004
Days logged into the EHR	217 (83)	218 (72)	263 (48)	248 (44)	.001
Appointments per scheduled day	8 (3)	9 (4)	6 (2)	7 (4)	<.001
Scheduled hours per scheduled day	4 (2)	6 (4)	3 (1)	4 (1)	<.001
Clinical preparation and follow-up					
Minutes in clinical review per appointment	6 (3)	6 (4)	8 (4)	6 (5)	.01
Minutes on orders per appointment	3 (1)	3 (2)	6 (2)	4 (2)	<.001
Electronic communication					
In Basket messages received per logged in day	16 (10)	27 (13)	24 (14)	23 (14)	<.001
Seconds per completed In Basket message	36 (27)	43 (36)	56 (28)	50 (27)	.003
Minutes on In Basket messages per appointment	3 (2)	5 (4)	8 (4)	6 (3)	<.001
Turnaround time per In Basket message (days)	5 (8)	5 (4)	6 (6)	5 (9)	>.99
Secure Chat messages sent per appointment	1 (1)	1 (1)	2 (2)	1 (1)	<.001
Work outside of work					
Minutes working outside of 7 AM-7 PM per scheduled day	20 (15)	22 (14)	25(17)	26 (20)	.20
Minutes outside scheduled hours per scheduled day	36 (27)	33 (27)	62 (31)	56 (42)	<.001

a Electronic health record.

b Analysis of variance.

**Figure 1.**
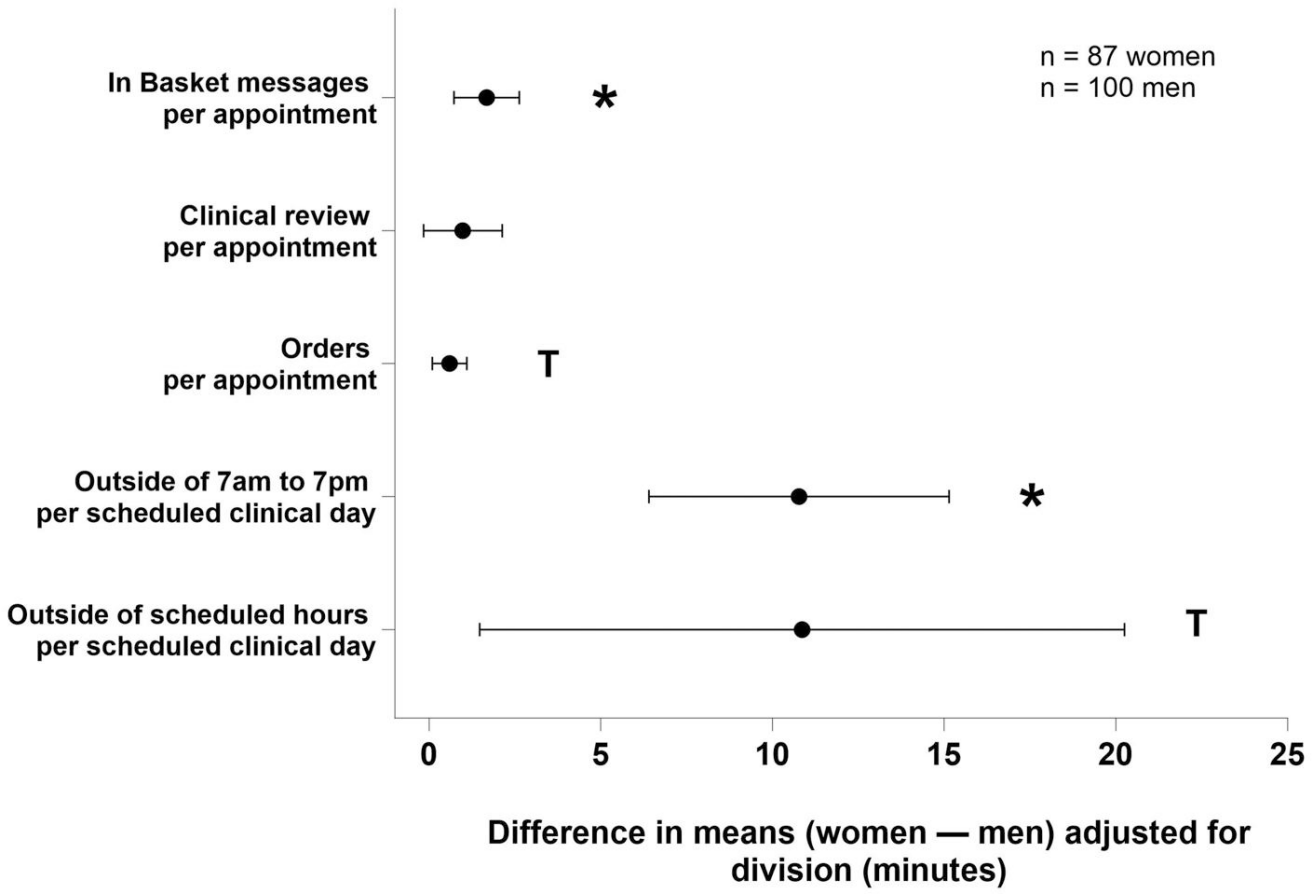
Time spent working in the EHR. Data are shown as adjusted means with 95% confidence intervals. *P < .004 (0.05/14 comparisons), ^T^*P* < .05. Abbreviations: CI, confidence interval; EHR, electronic health record.

**Figure 2.**
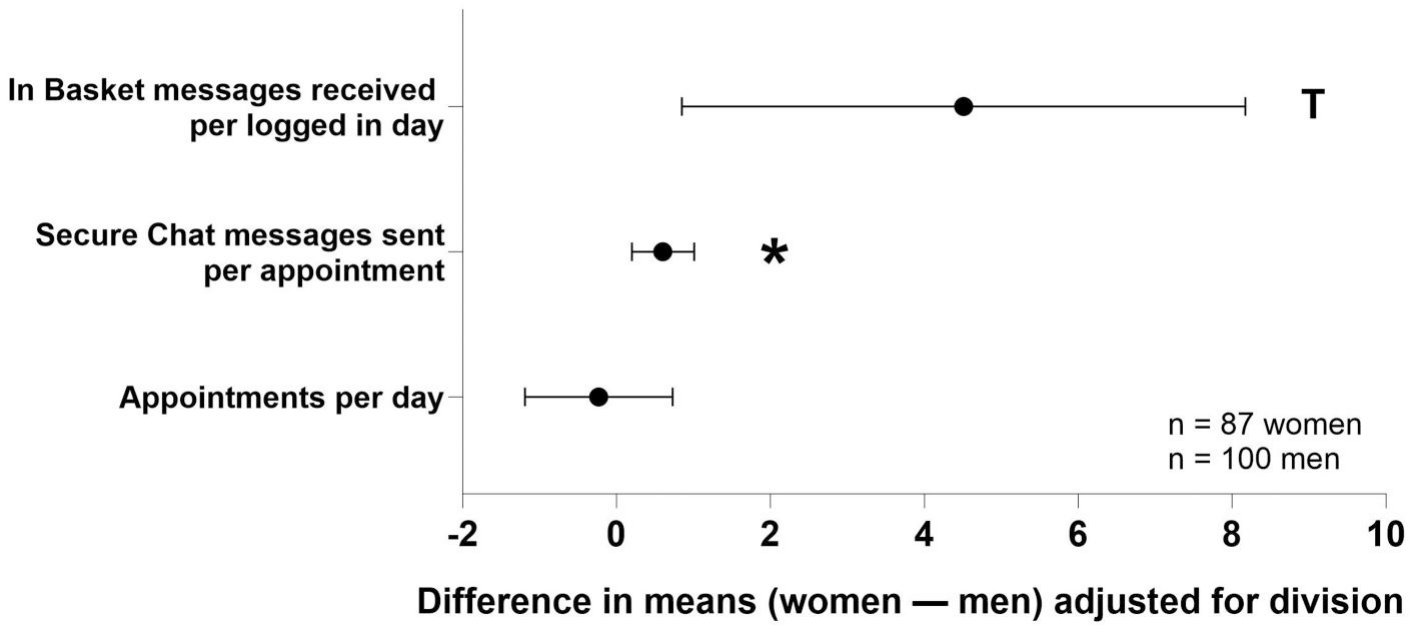
The number of EHR communications. Data are shown as adjusted means with 95% confidence intervals. *P < .004 (0.05/14 comparisons), ^T^*P* < .05. Abbreviations: CI, confidence interval; EHR, electronic health record.

The scheduled clinical workload (including the number of appointments) and the number of days a physician spent logged into the EHR did not differ by physician gender after controlling for medical specialty (Table 2). However, women physicians had a higher EHR workload than men by several metrics after adjusting for specialty and the number of comparisons (Table 3). More specifically, women spent more total time working on In Basket messages per appointment (*P*=.001, Figure 1) and sent more Secure Chat messages per appointment (*P* =.003, Figure 2) than men. Women and men physicians also differed in when they worked in the EHR. Women worked more time outside the 7:00 AM-7:00 PM period than men (*P* <.001; Figure 1). The number of In Basket messages received per day, time spent working on orders, and EHR work outside of scheduled hours did not statistically differ between women and men physicians when accounting for multiple comparisons. Finally, the time spent working on each In Basket message and the turnaround response time did not differ by physician gender (Table 3).

**Table 2.** Clinical workload by physician gender.

	Women (*n*=87)	Men (*n*=100)	Difference	*P*-value^a^
	Adjusted mean, 95% CI	Adjusted mean, 95% CI		
Per scheduled clinic day				
Number of appointments	7.4 (5.2 to 9.6)	7.6 (6.4 to 8.9)	−0.23 (−1.2 to 0.7)	.64
Scheduled clinical hours	4.4 (2.5 to 6.2)	4.4 (3.2 to 5.7)	−0.05 (−0.7 to 0.6)	.87
During study period				
Number of appointments	691.1 (423.4 to 958.8)	640.9 (532.3 to 749.4)	50.2 (−109 to 209.4)	.54
Number of scheduled clinic days	96.5 (64.9 to 128.1)	86.9 (71 to 102.8)	9.57 (−6.1 to 25.3)	.23
Number of days logged into the her	236.3 (193.6 to 279)	235.5 (212.9 to 258)	0.85 (−19.3 to 21)	.93

Abbreviations: CI, confidence interval; EHR, electronic health record.

a Mixed-effects linear regression adjusted for specialty.

**Table 3.** EHR workload among women and men physicians.

	Women	Men	Difference	*P*-value^a^
	Adjusted mean, 95% CI	Adjusted mean, 95% CI		
Minutes in In Basket per appointment	6.4 (3.8 to 9)	4.7 (3.1 to 6.4)	1.7 (0.7 to 2.6)	.001*
In Basket messages received per logged in day	24.4 (16.6 to 32.1)	19.9 (15.8 to 24)	4.5 (0.9 to 8.2)	.02**
Seconds per completed In Basket message	45.6 (27.9 to 63.3)	46.4 (37.4 to 55.4)	−0.8 (−9.5 to 7.9)	.86
Turnaround time (days per In Basket message)	5.4 (2.1 to 8.7)	5.4 (4 to 6.7)	0 (−2.0 to 2.0)	.99
Secure Chat messages sent per appointment	1.5 (0.5 to 2.5)	0.9 (0.3 to 1.5)	0.6 (0.2 to 1)	.003*
Minutes in orders per appointment	4.0 (2.2 to 5.8)	3.4 (2.2 to 4.7)	0.6 (0.1 to 1.1)	.02**
Minutes in clinical review per appointment	7.0 (5.0 to 9.0)	6.0 (5.1 to 6.9)	1.0 (−0.2 to 2.1)	.09
Minutes outside of 7 AM-7 PM per scheduled day	28.4 (21.1 to 35.8)	17.6 (14.7 to 20.6)	10.8 (6.4 to 15.1)	<.001*
Minutes outside scheduled hours per scheduled day	51.7 (29.9 to 73.5)	40.8 (28.4 to 53.2)	10.9 (1.5 to 20.2)	.02**

Abbreviations: CI, confidence interval; EHR, electronic health record.

a Mixed-effects linear regression adjusted for specialty.

* *P* <.004 (0.05/14 comparisons).

** *P*<.05.

Specific gender differences were observed in data stratified by procedural and nonprocedural specialties. Among the procedural physicians, women spent more time working on In Basket messages per appointment than men (women: mean 5.3 [SD: 3.2], men: 2.9 [2.3], *P*<.001, *d* = 0.89). Women also sent more Secure Chat messages per appointment (women: 1.3 [1.0], men: 0.7 [0.9], *P*=.007, *d* = 0.53). Additionally, the women spent more time working in the EHR outside of 7:00 AM-7:00 PM than men (women: 24.2 [15.3], men: 18.3 [13.4], *P*=.037, *d* = 0.41).

For the nonprocedural physicians, women spent time working in the EHR outside of 7:00 AM-7:00 PM than men (women: 32.6 [17.6], men: 16.3 [13.6], *P*<.001, *d* = 1.02). However, there were no gender differences in time spent working on In Basket messages (women: 7.8 [3.9], men: 6.6 [3.5], *P*=.197, *d* = 0.41) or number of Secure Chat messages sent (women: 1.9 [2.0], men: 1.3 [1.8], *P*=.142, *d* = 0.32) per appointment.

## Discussion

Evaluating physician workload in the EHR is essential to identify workflow changes that could improve job satisfaction, support retention, and mitigate further staffing shortages in medicine.^1^^,^^2^ When comparing EHR workload in 4 medical specialties within ambulatory settings, we found no differences in the scheduled clinical workload by physician gender. However, compared to men, women physicians spent significantly more time communicating with patients, other clinicians, and staff through In Basket messages. Women also sent more Secure Chat messages to other clinicians and staff. Altogether, this additional EHR work likely contributed to women physicians spending more time working in the EHR outside of 7:00 AM-7:00 PM than men. When separately examining procedural and nonprocedural specialties, the greater message workload for women was specifically concentrated in the procedural specialties. Thus, different approaches to mitigate the EHR burden for women physicians may be needed for procedural and nonprocedural specialties.

Our study adds to a growing body of literature^21–25^^,^^30^ demonstrating a disproportionately high EHR workload for women physicians. Thus, physician gender differences in the EHR burden may be a widespread problem. Health-care leaders nationwide should consider strategies to streamline EHR workflow for all clinicians, especially women physicians. Studies have shown that women have similar^24^ or even increased^23^^,^^31^ efficiency in using the EHR relative to men. We found that the time spent working on each In Basket message and the turnaround response time did not differ by physician gender. Thus, our data also do not indicate that women are less adept than men with the EHR.

The finding that only women procedural physicians sent more and spent more time on messages to patients, other clinicians, and staff than men physicians warrants further study. This gender difference in messaging did not occur in the nonprocedural specialties. Differences in EHR use have also been reported for surgical and nonsurgical clinicians.^32^ A potential contributing factor is the additional preparatory and follow-up work for diagnostic and interventional procedural suites. This includes arranging schedules for the procedures, coordinating the procedural clinical teams and equipment before, during, and after the procedures, and answering patient questions about preparing for the procedure and the results. While all procedural physicians conduct these communications, a greater messaging burden may fall upon women. Patients may ask more questions and seek more in-depth answers before and after a procedure from women physicians.^33^ Moreover, there may be different distribution and quality of clinical and administrative support between procedural and nonprocedural specialties at our institution. While we could not examine this here, it is also possible that women physicians rely less upon administrative support than do men if their work culture includes gender bias and microaggressions against women.^34^ Women would consequently have to move their EHR work to the evenings. Future qualitative studies could provide detailed information on the role of such social contributors to gender differences in EHR use.

High EHR workloads contribute to clinician burnout.^6^ In our study, women spent approximately 11 more minutes working in the EHR outside of 7:00 AM-7:00 PM per day than men. Assuming 20 clinical days per month, this additional work translates to more than or 43 h per year of additional EHR time for women. In addition to lost opportunities for research, teaching, writing, lecturing, and time for well-being, this extra time may represent uncompensated work that exacerbates the gender wage gap, estimated to be 2 million dollars less income for women physicians over their careers.^35^^,^^36^

Societal expectations for gendered family and household responsibilities may influence when women physicians can do their EHR work. Jolly et al.^37^ reported that women physicians are more likely to have partners who are employed full time, whereas men are more likely to have a partner who stays home or works only part-time. Women physicians with children spend an estimated 8.5 more hours per week on domestic activities than their spouses or partners. Thus, many women physicians may only find time for EHR work in the late evenings after they finish their family and household duties.

The greater time that women procedural physicians spent on messaging in our study could reflect differences in patient communication and coordination of care. Since patients tend to disclose more information to women providers,^38^ they might make more complex requests of women physicians. Women physicians may also spend more time answering their patients in highly patient-centered care.^39^ Moreover, women physicians may also have more women patients^24^^,^^40^ who have more participatory visits than men patients.^41^ We could not evaluate the content of the messages, and future studies are needed to determine whether there is an association between messaging and clinical outcomes. Some studies suggest better outcomes from women physicians,^42^^,^^43^ whereas others show no effect of physician gender.^44^^,^^45^

Strategies to alleviate the physician EHR workload include transitioning toward team-based staffing models for EHR communication. This would require systematic efforts to ensure that women and men physicians have the same amount of qualified personnel resources, including nurses and support staff, who can conduct pre- and postvisit planning and answer questions independently of the physician. The EHR could be optimized to triage communications directly to the appropriate clinical team member and bypass the physician. Ideally, physicians should only see an EHR message if they specifically need to respond. Future research should explore how artificial intelligence can streamline EHR communications and team-based staffing models, including direct correspondence with patients, other clinicians, and staff. Future studies may also consider qualitative methods with patients and providers to better understand communication thresholds and barriers.

The interactions between physician gender, specialty, and workload must be examined in large multi-institutional studies as different workflows, staffing, culture, social norms, and other factors may influence the results. We examined 4 ambulatory specialties in our study. In reality, numerous specialties at our institution may have this problem, but we have not yet examined each one. The influence of artificial intelligence applications to the EHR should also be studied to explore how such technology affects the gendered EHR burden.

Our findings are subject to limitations. Our data were limited to one academic medical institution, and we did not broaden the retrospective study period to avoid confounding effects from the COVID-19 pandemic. While multiple covariates were considered, we might not have accounted for hospital-level or region-specific confounders affecting physician practice patterns. Our analysis was limited to variables available in Epic Signal. Thus, we did not adjust the analysis for patient complexity or administrative support. Nonetheless, these factors should be taken into consideration with future studies. We selected participants from 4 ambulatory settings, and the findings may not be generalizable to physicians working in inpatient settings or other specialties. Finally, we could not measure the content or quality of the EHR communications, the message source, or demographic details about the patients such as gender.

## Conclusion

Women physicians in academic ambulatory settings spent more time on messaging with patients, clinicians, and staff and working in the EHR outside of 7:00 PM-7:00 PM than men physicians. The greater messaging workload for women was concentrated in procedural specialties. Creating a more equitable work environment will require improving how the EHR is used in a physician’s workflow, managing any gendered expectations from patients and colleagues, and optimizing clinical resources equally among physicians of all genders.

## Data Availability

The data underlying this article will be shared on reasonable request to the corresponding author.
